# Treatment of eating disorders in adolescents during the COVID-19 pandemic: a case series

**DOI:** 10.1186/s40337-021-00374-z

**Published:** 2021-02-10

**Authors:** Serur Yaffa, Enoch-Levy Adi, Pessach Itai, Joffe-Milstein Marit, Gothelf Doron, Stein Daniel

**Affiliations:** 1 Pediatric Psychosomatic Department, Sheba Medical Center, 5265601 Tel Hashomer, Israel; 2 Child and Adolescent Psychiatric Division, Sheba Medical Center, 5265601 Tel Hashomer, Israel; 3 Edmond and Lily Safra Children’s Hospital, Sheba Medical Center, 5265601 Tel Hashomer, Israel; 4 Sackler Faculty of Medicine, Tel Aviv University, 69978 Tel Aviv, Israel; 5 Sagol School of Neuroscience, Tel Aviv University, 69978 Tel Aviv, Israel

**Keywords:** COVID-19, Eating disorders, Pandemic, Telemedicine

## Abstract

**Abstract:**

**Background:**

Eating disorders (EDs) are among the most difficult psychiatric disorders to treat in normal conditions. They are likely even more difficult to manage in at-risk conditions such as the COVID-19 pandemic. Currently there is limited evidence about the particular needs and recommended treatment of adolescents with EDs during the COVID-19 outbreak, in particular regarding the use of telemedicine and the involvement of the family in long distance-treatment.

**Aims:**

We sought to discuss the advantages and problems associated with the use of multi-professional long-distance telemedicine treatment in the management of adolescents with EDs and their families during the COVID-19 outbreak.

**Methods:**

We gathered data about the treatment of adolescents with EDs in our pediatric ED-treatment center in Israel during the COVID-19 outbreak in the first 10 months of 2020, and compared it to the respective period in the past five years (2015–2019). Second, we described the management of four young females with anorexia nervosa (AN), treated in the ambulatory, daycare and inpatient facilities of our center during the COVID-19 pandemic.

**Findings:**

Slightly less patients were treated in our center during the COVID-19 pandemic than in the respective period in the past five years. These patients received at that time considerably more treatment sessions from all treatment providers (psychiatrists, clinical nutritionists and psychotherapists). This was related, in part, to the extensive use of telemedicine during that period (more than as third of all sessions were carried out with telemedicine in comparison to no use of long-distance treatment in the previous years). The condition of the four adolescents with AN was compromised at the start of the COVID-19 quarantine. The use of multi-disciplinary long-distance telemedicine treatment resulted in an improvement in the condition in three of the four adolescents, living in well-organized families, with the motivation and ability to adjust to the new conditions, but not in one adolescent whose family experienced more problems. These families might require the use of face-to-face interventions even during pandemic conditions.

**Conclusion:**

The choice of the mode of treatment for adolescents with EDs during pandemic times (telemedicine vs. face-to-face) should consider the functioning of the family.

## Lay summary

Anorexia nervosa is one of the most complicated psychiatric disorders to treat in normal conditions. It is likely even more difficult to manage in at-risk conditions such as the recent COVID-19 (Corona) outbreak. In this article we describe the management of four young female adolescents with anorexia nervosa during the COVID-19 quarantine in a center treating children and adolescents with eating disorders in Israel. We used long-distance treatment (telemedicine) with a team of dietitians, psychotherapists and psychiatrists to help the parents and patients with eating at home, and with other problems arising from the COVID-19 outbreak and the associated changes in living conditions. The findings show a deterioration in the condition of the four adolescents at the start of the COVID-19 quarantine. They found it difficult to adjust themselves to the greater involvement of their families with their eating, and to the reduction in physical activity because of the required confinement. The use of multi-professional long-distance treatment showed an improvement in the condition of three of the four adolescents, living in well-organized families, with the motivation and ability to adjust to the new conditions, but not for one girl, whose family experienced more problems.

## Background

In March 2020, the World Health Organization (WHO) declared the Corona Virus (COVID-19) outbreak in 2020 as a pandemic, affecting economy, healthcare, and well-being. Due to the need for social distancing, in-person visits for clinical care were reduced worldwide, and treatment providers had to learn new skills within a brief period and adapt clinical care to the new conditions [1, 2].

People with psychiatric disorders are affected significantly more by external stressors such as the COVID-19 outbreak, likely due to them using more dysfunctional emotion regulation and coping strategies than healthy individuals [3]. Adults [4, 5] and adolescents [6] with psychiatric problems have been found to show acute emotional reactions to the COVID-19 outbreak, with its consequent quarantine and social isolation, including depressive, anxiety, and trauma-related symptoms [4–6].

### EDs in the time of COVID-19

Anorexia nervosa (AN) is one of the most complicated psychiatric disorders to treat in normal conditions [7]. It is likely even more difficult to manage in at-risk conditions such as the COVID-19 pandemic. This is because the management of these patients usually requires regular clinical follow-ups. However, during the current COVID-19 pandemic, face-to-face clinical assessments carry a particularly high risk of infection and transmission in this vulnerable cohort, already compromised by low weight and reduced immunity [8, 9].

Recent surveys of adult patients with AN and bulimia nervosa (BN) [4–7, 10–15] during the COVID-19 pandemic found an increase in eating disorder (ED)-related symptoms, heightened overall anxiety, and reduced quality of life. Patients reported of an increase of 39–87% in the frequency of food restriction, binge-eating episodes, and purging behaviors. They also reported fears of loss of routine and control regarding their meal plan, related in part to reduced access to ED treatment services. The provision of adequate care in patients with EDs during periods of acute pandemic and confinement requires both psychological and medical interventions in people who might be medically compromised, resistant to treatment, and suicidal [9, 15–17]. These interventions have to be relatively easy to implement, and not necessarily carried out with face-to-face meetings. They require adaptations for dealing with issues such as home weighing and management of binge/purge behaviors, likely involving multiple interventions from different treatment providers with different family members to adequately supervise eating.

In contrast to the findings in adult patients, there is only limited information about the treatment of adolescent patients with EDs during the COVID-19 outbreak [9, 18, 19]. A recent study [9] in seven adolescent patients with AN suggested a treatment program tailored specifically for the COVID-19 period. The program included long-distance home-based sessions instead of clinic/hospital attendances, biweekly weighing, recording of food intake to allow for estimated calculations of daily calories, estimation of energy expenditure, and collection of vital signs when possible. The clinicians in this study used a structured clinical interview with the parents to make accurate clinical assessments remotely. While there was no description of the efficacy of this program, the authors emphasized its potential utility in maintaining ongoing delivery of treatment and parental support, balancing the protective effect of few or no clinical attendances against the risk of excessive carer burden and unnoticed patient deterioration. In another study [19] carried out during the COVID-19 outbreak, almost half of the children and adolescents with EDs studied, experienced reactivation of their ED symptoms despite treatment, and symptoms of self-harm and increased suicide risk occurred in patients with severe illness (25% of the sample). In that study, telephone and videoconferencing visits were held in both the day hospital and outpatient programs; under this program, certain face-to-face visits continued, while combined treatment was instituted according to clinical indication.

### Telemedicine treatment in the time of COVID-19

The findings of previous studies about the treatment of patients with EDs during the COVID-19 period [9, 19] highlight the importance of adapting to the use of long-distance telemedicine. Telemedicine refers to the provision of remote clinical services, via real-time communication, between patients/families and healthcare providers, using electronic audio and visual means. Telemedicine services may expand access by reducing barriers such as travel time, competing responsibilities, or absence from work, and provide advantages for treatment providers and institutions, including schedule flexibility, increased productivity, and less clinic overhead [2]. With the ongoing COVID-19 pandemic, telemedicine might be also useful in decreasing emergency room visits and safeguarding healthcare resources, potentially reducing the spread of the virus [20].

Moreover, no difference was found in the effectiveness and treatment adherence of face-to-face vs. internet-based interventions in people with mental-health problems [21–24]. Specifically, videoconferencing was as effective as in-person treatment for reducing the severity of post-traumatic, anxiety and depressive symptoms [25–27]. Similarly, cognitive behavioral treatment (CBT) by videoconferencing (telehealth) had good clinical efficacy for ED treatment [28], including in adolescents [29]. Moreover, telehealth was found appropriate also during the COVID-19 pandemic in reducing the mental health burden of patients and families [30–33]. For example, studies comparing telemedicine to face-to-face interventions during the COVID-19 outbreak found positive experiences with both options in caregivers of adult patients with EDs [34]. Last, psychologists who were more likely to use teletherapy prior to COVID-19, being more comfortable with its use, showed more positive attitudes to telepsychology, and in turn, greater use of it, during the pandemic [35].

### The aims of the present study

Given the scarcity of data regarding how to support adolescents with EDs during the COVID-19 pandemic, and the need to adapt treatment to the specific conditions of the resulting mandatory confinement, we sought to analyze our data about the management of adolescent patients with EDs in Israel during the COVID-19 period. Second, we sought to describe four female adolescents with AN treated in the different settings of our pediatric ED-center, emphasizing the obstacles and challenges of the treatment of EDs under these conditions. The four cases described here illustrated the emotional condition of adolescents with AN during the COVID-19 outbreak, the way in which they and their families adapted to the quarantine, and the pros and cons of telemedicine use.

## Methods

### Treatment provision in the present study

The pediatric ED treatment center in the Safra Children’s Hospital at the Sheba Medical Center, Tel Hashomer, Israel, includes inpatient, daycare, and ambulatory services for children and adolescents between the ages of 6–18 with diverse types of EDs. Treatment is provided by a multidisciplinary team at varying levels of intensity, depending on the severity of the ED and comorbid disorders, and the overall functioning of the patient and family. The center is a “wrap-around” service, i.e., patients may move from one facility to another, depending on their condition.

During inpatient treatment, patients receive treatment interventions tailored for the treatment of the ED, comorbid disorders, and different psychosocial difficulties. Upon discharge, patients are offered a multidisciplinary daycare program in the afternoon hours, to allow for their reintegration into the school system. When considered stabilized, patients are referred to our ambulatory service. Most of the patients in our ambulatory facility are sent from different community services or are self-referred.

The integrative treatment protocol in our service corresponds with other structured programs for adolescents with AN and BN [36, 37]. The protocol includes the following: a behaviorally oriented nutritional rehabilitation program; either individual psychodynamic-oriented psychotherapy or individual CBT, depending on the specific illness and the aims of treatment; individual expressive movement therapy; family therapy (either family-based treatment (FBT [38]) or systemic family therapies [39]) or parental consultation; and different group interventions: psychodynamic group therapy, CBT group sessions (“classical” ED-related CBT [40] and cognitive-motivational treatment based on the Maudsley Model of Anorexia Nervosa Treatment for Adults (MANTRA) protocol [41], and group expressive movement therapy. The inclusion of psychodynamic psychotherapy in the treatment regimen is designed to address intrapsychic and interpersonal developmental needs of adolescents often burdened with long-standing illness, in addition to the specific ED-related therapies administered [42]. It is of note that other programs in patients with AN have used psychodynamic psychotherapy as their main treatment, showing favorable results [43]. While inpatient treatment includes all these therapeutic modalities, daycare and ambulatory treatment includes only some of these interventions, as required, but nutritional consultation, individual psychotherapy, family therapy/parental consultation, and some group interventions are usually maintained.

### Data analysis

For this study, we have retrospectively reviewed data about the treatment in our ambulatory service during the COVID-19 period. This data appears in a specific computerized database of the Sheba Medical Center called CHESS. This database includes data about the number of patients, sessions, and treatment providers treating the patients (in our case psychiatrists, clinical nutritionists and psychotherapists) in a certain period. It does not include any other data. We further describe the management of four female adolescents with AN treated in our ambulatory, daycare and inpatient services.

### Findings

The first cases of COVID-19 in Israel were reported in February 2020. Starting on March 15th, 2020, and continuing for two months, people were not allowed to be treated frontally unless for emergency care; this required the transfer of most of our services to telemedicine to provide patients and families with adequate support and continuity of clinical care. In the ambulatory service, psychiatric, nutritional and psychological treatment were aimed to be continued as before with specific required adaptations, but carried out via telemedicine. Individual, family therapy/parental counseling and group therapies were, thus, offered online. Nutritionists asked the patients to weigh themselves weekly with the supervision of their parents, if this was possible and agreed upon by both parties. Weighing took place during the morning hours at the patients’ home or at the nearest community medical service. Patients were also asked to carry out regular food monitoring. Face-to-face visits occurred only in specific conditions.

All daycare patients, and those inpatients considered physically and emotionally stable, were discharged to their homes. There, they received a long-distance treatment protocol similar to that of the ambulatory services, but were asked to measure weight and vital signs once a week at the hospital (similar to the recommendations of other studies, e.g., Graell et al. [19]). Patients continuing with inpatient treatment were not able to leave the hospital during the two-month COVID-19 lockdown, or to receive visits from their families and peers. Individual sessions with the multidisciplinary treatment providers still continued on a face-to-face basis, but all family treatment/parental consultation sessions were transformed to telemedicine (with the patients and therapists in the department, and the parents and other family members at home).

Telemedicine services enabled maintenance of continuity of care until the end of the quarantine, when face-to-face services could be resumed. Thereafter, some of the ambulatory and daycare treatments continued with telemedicine because of the confinement of patients, families, or treatment providers, or because of the refusal of patients/families to be managed frontally in a hospital treating patients with COVID-19. Other patients and families resumed face-to-face interventions.

A second quarantine occurred between the 13th of September and the 11th of October 2020. Ambulatory, daycare and stabilized inpatients were again offered home-based long-distance telemedicine treatment, but in contrast to the first lockdown, the decision of keeping the treatment face-to-face vs. via telemedicine could be agreed upon by treatment providers and/patients/families. It should be noted that although possible, we did not treat our patients with telemedicine prior to the COVID-19 outbreak.

The findings emerging from the treatment in our ambulatory service during the COVID-19 pandemic between 01/01/2020–31/10/2020 have been unexpected. Table 1 summarizes the findings for the 2.5 months before the COVID-19 outbreak (01/01/2020–15/03/2020), the 2 months of the first COVID-19-related lockdown (15/03/2020–15/05/2020), the 4 months after the release of the 1st mandatory quarantine (15/05/2020–13/09/2020), the four weeks of the second mandatory quarantine (13/09/2020–11/10/2020), and the next three weeks until 31/10.2020. We have compared this data to the respective period in 2015–2019 (see Table 1).

**Table 1:** Data of treatment in our ambulatory service during the COVID-19 pandemic and the mean respective period between 2015 and 2019

	2020 (01/01–31/10)	Mean yearly 01/01–31/10; from 2015 to 2019
**Sessions**	5926	4001
**Patients**	242	257
**New patients**	127	166
**Meeting with a psychiatrist**	662	433
**Meeting with a clinical nutritionist**	1722	750
**Meeting with a mental health professional (psychologist, art/movement therapist)**	3318	1690
**Telemedicine Sessions (with any team member)**	2192	_______

The findings show that for the period of 01/01–30/10, ambulatory patients and their families have received more multidisciplinary sessions during the time of the COVID-19 (5926 sessions), compared to the respective period between 2015 and 2019 (4001 sessions) from all team members - psychiatrists, clinical nutritionists, and psychotherapists. The number of patients treated in our ambulatory service during these ten months was slightly lower than their mean yearly number in the respective previous period (242 vs. 257); although fewer new cases were accepted at that time, their number was still considerably high. The increase in the number of sessions was accounted for, at least in part, to the possibility of carrying-out multi-professional telemedicine meetings, comprising of 37% of all sessions during the first 10 months of 2020, vs. no use during the respective period between 2015 and 2019 (2192 of 5926 sessions, see Table 1).

### Case reports

Due to the nature of this case-report study, ethics approval was not required by the institution. Demographic and clinical details of the participants were changed to prevent identification. Verbal consent was obtained for publication from patients and parents.

### Ambulatory treatment: case 1

M. is a 13-year old 7th grade female student, the youngest of three children of divorced parents. There is no evidence of psychiatric history in the family. M. is living with her mother, but visits her father regularly. She is diagnosed with attention deficit/hyperactivity disorder, learning disorders, and generalized anxiety disorder.

M. was referred to our ambulatory service following a short hospitalization in a pediatric department because of severe bradycardia (HR at night ≤44 BPM). She was diagnosed with restricting type AN and a major depressive episode with occasional suicidal thoughts and severe self-injurious behaviors (SIBs). At admission to our service, her body mass index (BMI) was 16.9 kg/m^2^. M. started with fluoxetine up to 30 mg/day and risperidone up to 1.4 mg/day. The treatment was via telemedicine from the start, and included weekly meetings with a clinical nutritionist, psychiatrist and psychologist. She was weighed at home in the presence of her mother. M. was afraid to gain weight and reacted with impulsive SIBs, requiring the presence and support of the nutritionist and psychiatrist via telemedicine during the process of weighing. Directly after weighing, M. had a telemedicine session with her psychologist to help her cope with her anxiety. Additionally, because of the COVID-19 quarantine, her older siblings returned home. Her sister was constantly exercising, and both siblings were supervising and criticizing M’s eating, making her angry and less cooperative. Telemedicine sessions enabled flexible treatment settings, which met with M.’s changing needs and abilities and enabled the inclusion of the family to assist her. The siblings were invited to participate in the telemedicine sessions with M. and the psychologist, thus reducing their criticism and increasing their cooperation with the treatment. Sessions with the mother empowered her supervision over her eating.

M. reached the minimal range of her target weight within two months, followed by the return of her menses. She gradually returned to school and meet with her friends. She showed good control over her eating, and her psychotherapy concentrated mainly on the problems she had with her peers. Upon the request of M and her mother, individual psychotherapy has been resumed on a face-to-face basis, whereas the consultations with the mother (with a different psychologist), family sessions with different family members (carried out by the two psychologists), nutritional consultation (including weighing), and psychiatric consultation (she is currently treated with duloxetine 30 mg/day and risperdone 1 mg/day) are carried out with telemedicine.

### Ambulatory treatment: case 2

N. is a 14 years-old, 9th grade female student, the third of four children, living with her parents and sisters. There is no known family history of psychiatric disorders. Her parents report normal development.

She was diagnosed with AN-purging type with additional rage outbursts, including shouting and the throwing of objects. N. was referred to our ambulatory service following a hospitalization in a pediatric department because of low weight (BMI = 15.4 kg/m^2^) and lack of cooperation with her previous ambulatory treatment. During this hospitalization, she was treated with risperidone 0.5 mg/day, with a decrease in the frequency and intensity of the outbursts.

When school was discontinued because of the COVID-19 outbreak, all the family was confined at home. N.’s severe AN-related eating behaviors were brought to the foreground. She ate very slowly, spat out her food and was frequently in the toilet following her meals. The sessions with her psychiatrist, nutritionist and psychologist were transferred to telemedicine. She met with the psychiatrist every two weeks, and had a once-weekly session with her psychologist, and once- weekly session with the nutritionist. The mother was present with the girl in several nutritional counseling sessions, but not in others. In the sessions where only N. was present, she reported of feeling better, denying all her ED-related symptoms, and constantly demanded the stopping of her medications. When the nutritionist heard about the worsening of her symptoms in the sessions where the mother was also present, she recommended a complete supervision of N.s’ meals, including supervision on the preparation, eating and avoiding her entering the restroom after meals. As the father continued working outside the home, and the mother was confined to bed because of severe back pain, it was decided by the team and parents that N’s sisters would cook and supervise her meals. The mother felt helpless in this position and asked for more support from the treatment team. As a result, in addition to the regular telemedicine treatment plan, we included a once-weekly separate guidance to the mother.

Our plan was that N. would benefit from the support of all her family at home during the COVID-19 quarantine to rehabilitate her eating. In this case, however, the opposite occurred. The family intervention brought to continuous tensions between N. and her mother and sisters, and N’s rage outbursts worsened again. She resented her sisters for telling her what to do, and the mother was feeling helpless despite her separate supervision. The long-distance telemedicine family consultations and individual psychotherapy sessions had no effect on N.’s outbursts and her cooperation with her meal plan.

After gaining one kilogram (BMI = 17.6 kgs/m^2^), N. refused any supervision on her eating, stopped cooperating with the team, and started to lose weight. The family felt that they could not handle N.’s supervision anymore. After the first quarantine ended, N. was admitted to inpatient treatment; currently, she is still hospitalized.

### Daycare treatment: case 3

Y. is a 15 years-old 10th grade female student, diagnosed with AN-restricting type, the second of four children, living with her parents and siblings. There is no known family history of psychiatric disorders. There have been no reported problems during her development.

Y. was referred to our half-way out daycare service following seven months of inpatient treatment, in which she was cooperative and was able to reach her recommended weight. The transition from inpatient treatment to daycare was very hard for Y. She struggled with eating independently, leading to gradual but constant weight loss and irritability. The multidisciplinary treatment schedule in the day-care center included psychological treatment, nutritional and psychiatric supervision, parental counseling, and various group therapies carried out in the hospital setting three times a week. To prevent re-hospitalization, Y.s’ parents were encouraged to increase their involvement with her eating at home.

At the break of the COVID-19 pandemic, Y. was on the waiting list for recurrent inpatient treatment. At that time, all the daycare services had to be changed to telemedicine, including her once-weekly psychiatric, nutritional, psychological and parental counseling. Group therapy with other patients from the daycare treatment was also performed via telemedicine once a week. Y. was weighed once weekly in the hospital, this being the only face-to-face intervention. She continued with daily food monitoring with the assistance of her parents.

As Y. was at home with her family, this allowed for the augmentation of family interventions. The aim of this treatment was to use the opportunity of the COVID-19 confinement to improve the communication between Y. and her parents, and to empower her parents in assisting her with her eating. The parents, themselves, were preoccupied during the lockdown with their own physical training and dieting. In the sessions with the psychologist, Y. shared that her parents could not assist with her eating because of their own physical activities and dieting, thus refusing to cooperate with their supervision. The parents’ behaviors were brought to the foreground and discussed in the telemedicine parental counseling, leading to their willingness to change them with the assistance of the treatment team. This, in turn, led Y. to agree to eat under her parents’ supervision, gradually gaining weight again, eventually reaching her target weight. After the COVID-19 contingencies of the first quarantine were removed, Y. was able to resume daycare treatment, nor requiring re-hospitalization anymore. Currently she is in ambulatory treatment, with all sessions carried out face-to-face.

### Inpatient treatment: case 4

S. is 17 years old, a 12th grade female student, the second of two children, living with her parents. There is a family history of anxiety disorders. She is diagnosed with social anxiety disorder from an early age.

S. developed AN-restricting type with comorbid major depressive disorder in the past three years. Combined with her pre-existing social anxiety disorder, she stopped attending school and later did not leave her room for prolonged periods. S. was hospitalized in our center after a year of unsuccessful ambulatory treatment for her ED. When the COVID-19 outbreak started, she was already hospitalized for four months. At that time, S. reached her target weight range and her depression was in remission. Although she was planned to be released gradually from inpatient treatment to daycare, the COVID-19 outbreak required her immediate discharge to virtual telemedicine home-based treatment. This treatment included once-weekly individual psychotherapy and once-weekly parental, nutritional and psychiatric counseling, alongside the provision of school services via telemedicine three days a week. S. was weighed every two weeks in the hospital by the department’s nurse. This new treatment program continued for three months.

Despite this intensive multi-professional treatment, the change of the treatment setting was followed by a continuous deterioration in S.’s condition. This included the returning of restriction of food intake and of previous maladaptive eating-related behaviors and social withdrawal. S. refused to take her medications (fluoxetine 20 mg/day and risperdone 1 mg/day) and shared with the staff that she had difficulties coping without the support of the inpatient setting. As a result, the team decided to augment the parental telemedicine counseling sessions to twice weekly and long-distance schooling to five days weekly.

These changes were possible because of the motivation of the treatment staff to be flexible and treat after the regular working hours. The flexible use of telemedicine with different team providers and different family members, at different times, enabled the carrying out of this plan. Indeed, the family assisted S. in reducing her avoidant strategies, and regain her studying – first in the department’s long-distance classes and thereafter, when the mandatory lockdown was released, to gradually return to her previous school, alongside an improvement in her eating behaviors and weight. Overall, the use of intensive home-based multidisciplinary telemedicine during the pandemic quarantine allowed for the integration of S. back into our daycare program and her school when the COVID-19 contingencies were released. Currently she continues with ambulatory treatment, performed totally with face-to-face interventions.

## Discussion

### Overall data

The COVID-19 outbreak in Israel has developed rapidly and unexpectedly from March 2020. The analysis of the treatment data in our ambulatory service during the first 10 months of 2020 shows greater use of mental health service during the COVID-19 outbreak and enforced quarantine in adolescents with EDs in comparison to regular times. The increase in the number of sessions has been likely accounted for, at least in part, by using multi-professional telemedicine meetings, comprising of 37% of all sessions during the first 10 months of 2020, vs. no use in the respective period in 2015–2019 (see Table 1). It is of importance to note, with respect to the considerable use of telemedicine in our study, that our team, like any other team in the hospital, has been instructed by specific professionals about the use of telemedicine, both technically and regarding its advantages and risks. This may have accounted for the overall positive reactions of most team members from all professions to using telemedicine in the treatment of adolescents with EDs, although being mostly not familiar with its use previously.

Our findings are in line with a study performed in the United States and Netherlands [10], showing that 42–45% of adult patients with EDs have transitioned to online/telehealth care during the COVID-19 pandemic. They are also similar to a recent study in adolescents with EDs in Spain [19], reporting more visits, but a lower number of patients, during the COVID-19 confinement compared with the same period in the previous year. In that study, the treatment team held 1329 (73.10%) telehealth consultations and 489 (26.9%) face-to-face outpatient visits with 365 patients undergoing ambulatory or daycare treatment. Nonetheless, another study of ambulatory adolescents with EDs in Singapore showed a decrease of 50% in treatment sessions during the COVID-19 period [18]. Our findings also stand in contrast to a survey of adult patients with BN in Germany, reporting that face-to-face psychotherapy decreased by 56% during the COVID-19 pandemic, and videoconferencing therapy was used only by 22% of the patients [13].

Several explanations can be suggested for these discrepancies. In contrast to other studies, telemedicine was not used in our service before the COVID-19 breakout. Second, in the first lockdown in Israel, everyone stayed at home, both parents and children. This could have increased tension and distress, and in turn, also ED-related symptoms, altogether requiring more intensive treatment [4–7, 10–15]. Thus, the COVI9–19 pandemic with its strict mandatory confinement might have created an environment that developed tougher-to treat EDs. This was highlighted in our study by the greater number of sessions administered at that time with only a slight decrease in the number of patients using them (see Table 1). At the same time, the provision of telemedicine, with all family members confined to their home, enabled this greater use of treatment. Overall, these conditions likely increased the cost of care, despite the cost of telemedicine being lower than that of face-to-face interventions.

Our data is similar, in this respect, to findings in Spain, another country requiring strict confinement at the start of the COVID-19 [19], leading to greater usage of long-distance treatment. Other telemedicine- associated factors contributing to the increase in the number of treatment sessions in our study can be related to the high flexibility in the scheduled sessions, and to less cancellation of meetings from patients and treatment providers.

### Case reports

The condition of our four female adolescents with AN has been compromised at the start of the COVID-19 quarantine. Changes in physical activity, sleep patterns, screen time and eating have been reported in healthy children and adolescents during periods without school, when spending most of the time at home [44]. These effects may be exacerbated when children are confined to their homes, causing them to have little contact with their peers and depriving them of outdoor activities [19]. However, the specific changes in the routine in adolescents with EDs, as described in our cases, seem to differ, showing an increase in physical activity rather than the decrease shown in healthy youngsters, and restricting rather than an increase in the amount of food eaten [19]. These changes in lifestyle may likely increase the risk of ED-related symptomatic deterioration [19], alongside the influence of acute depressive, anxiety, and trauma- related symptoms resulting from the COVID-19 outbreak, with its consequent quarantine and social isolation [4–6, 18].

In line with these findings, one study of adolescents with EDs treated in an ambulatory service in Singapore reported of an increase in health-related anxiety associated with the COVID-19 outbreak [18]. By contrast, our four adolescents hardly related to the pandemic by itself; they were mostly concerned with what was important for them personally [45]. Not surprisingly, this was associated with the influence of the quarantine-related change in their life conditions on their weight and eating/weight-related behaviors, as well as with the greater unwanted involvement of their families with their eating [see cases (1,), (2,), and 3).

Despite their seeming indifference to the direct health-related concerns of the pandemic, it is plausible to assume that because of their constricted, inflexible, rigid, and self-centered cognitive style [46, 47], our adolescents with AN may have been affected to a greater extent than healthy youngsters by the COVID-19 outbreak and its psychosocial consequences. Moreover, in adolescents with AN, the inability to escape the parents’ watching eyes at every meal during the confinement and to go outside and exercise, as described in the second ambulatory case, may increase their overall distress and distrust of others [47], adversely impacting their cooperation with treatment. Resulting fears of loss of personal control may trigger an increase in weight control behaviors in these adolescents to compensate for this loss [48]. This regression has been shown to occur, to some extent, in all four cases, although it could be improved in three of the four [Case (1, 3, 4)], following the intervention of the team with the eating-related involvement of their families.

Alternatively, youngsters with more severe AN and greater overall psychopathology [47], as described in Case (4), may have been isolated from their peers to some extent already before the COVID-19 pandemic. Along these lines, the absence during the COVID-19 quarantine in these youngsters of intense weight-related comparison driven by social contact [49], and the reduction in social stressors that hitherto increased their social-related anxieties [9, 49], might have actually reduced their overall distress. This has likely happened with Case (3) during her prolonged inpatient treatment, but not when staying at home during the COVID-19 quarantine, when losing the safe environment of the department.

### Telemedicine

Treatment of adolescents with psychiatric disorders in general, and EDs in particular, during the severe, unexpected changes of the COVID-19 and its associated quarantine, is highly challenging, requiring multidisciplinary collaboration, often with hitherto unfamiliar solutions. This is of importance, because in a recent study of adolescents with diverse psychiatric disturbances, most reported not only worsening of their mental condition during the COVID-19 pandemic, but a quarter stopped using mental health support [50]. This finding is very concerning, specifically for adolescents with EDs, who are often in need for multidisciplinary psychological, familial, nutritional and medical interventions [7].

In contrast to regular times, telemedicine in the COVID-19 period was not a matter of preferences by the patient/family or treatment providers. All treatment requirements, including weight measurements, had to be carried out online; all family members were at home, interfering with the patient’s privacy, the latter being especially important for adolescents with AN. In addition, families suffered from health- and economic-related preoccupations not present before [19, 30].

Several specific issues have to be considered regarding the telemedicine treatment of adolescents with EDs during the COVID-19 period. Thus, Rodgers et al. [51] have found that video conferencing in these patients may exacerbate body image concerns by increasing their preoccupation with and focusing on their self-appearance. In contrast, there is the issue of the “disembodied environment”, when only the patient’s and therapist’s faces, but not their bodies, appear on the screen, or when the patient prefers to converse in the session with the camera being closed. What is potentially missing in these cases is body-to-body communication, or the reading of body language [52].

In the present study we had the opportunity to learn from our own experience about the pros and cons of long-distance interventions in the treatment of adolescents with EDs. From the positive aspect, the telemedicine interventions were found to flexible, relatively easily and quickly arranged in times of crisis, and enabled the provision of treatment in hours that were more suitable for both treatment consumers and providers. Children did not have to leave school when the conditions of the confinement subsided, parents did not have to lose working hours, and other family members could relatively easily join treatment whenever required [see cases (1) and (2)]. Team meetings of different team members were relatively easily planned and carried out, including in the therapists’ homes. Moreover, the social distancing and “disembodied environment” generated with telemedicine was likely adequate for at least in three of the four cases [cases (1), (3) and (4)]. This is not surprising, bearing in mind the high rate of comorbid social anxieties in patients with EDs [53], and their concern with face-to-face emotional interactions, often considered by them to be overwhelming [54].

In these three cases, the families were quite well organized and flexible to work with telemedicine with the treatment providers according to the interventions recommended. In Case (1), this was despite the divorce of the parents, the lack of involvement of the father and both partners of the parents, and the unexpected return of the older siblings to the mother’s house because of the COVID-19 outbreak. The flexibility of the siblings in being able to change, following the team’s recommendation, their role from supervising the girl’s eating to empower the mother in her supervision, was of particular merit. In Case (3), the parents could change, with the assistance of the team, from being preoccupied with their own COVID-19 related problems to concentrate on the meal plan of their daughter. The augmentation of the parental telemedicine counseling sessions to twice weekly in Case (4) had a big influence on her cooperation with the parental supervised meal plan. These preliminary clinical findings suggest that adolescents with AN living in adaptive families can be managed with telemedicine in pandemic quarantine conditions. Nonetheless, it is of note that even in these favorable conditions, self-weighing, weighing in front of a family member, and meal supervision was difficult to implement.

Long-distance telemedicine consultation and psychotherapy was not adequate for Case (2), likely because of the lack of involvement of both parents with the treatment. The mother has been mostly confined to a helpless position, the father has not been involved, and telemedicine has not been enough to empower other family members (siblings). Our findings in this case, thus support the suggestion of Guo et al. [55], that families experiencing more problems may need more online intensive treatment or face-to face meetings with their treatment providers to assist their children. Interestingly, in this study [55], caregivers of patients with EDs have been found to show higher levels of depression and anxiety than non-ED caregivers, likely interfering with their potential to assist their patients. Moreover, a brief online education program has shown no effect on decreasing the depression and anxiety of the caregivers.

To summarize, the four cases described here highlight the important role of the family in improving, or alternatively maintaining, the adolescent’s illness [56], and in cooperating with the treatment of their daughter. This may become even more crucial in times of crises such as the COVID-19 quarantine, when the whole family is at home for prolonged periods, and has to be treated with long-distance interventions. Future studies of adolescents with EDs during pandemic times such as the COVID-19 outbreak, comparing between telemedicine, face-to-face and combined interventions with the use of systematic interventions and standardized assessment tools, should take into consideration the functioning of the family in the choice of the suitable mode of treatment for the specific adolescent and family.

## Conclusion

The condition of our four adolescents with AN was likely compromised at the start of the COVID-19 outbreak. Patients and families had to change their routines within a brief period and adjust to the new circumstances characterized by higher presence and greater involvement of family members with the adolescent’s eating, decrease in the options for physical activities, and lower access to treatment. The use of telemedicine treatment, which in our facilities was limited before the pandemic, offered new possibilities, including flexible multidisciplinary treatment provision with different treatment providers, the inclusion of different family members in different treatment sessions, and relatively easily planned team consultations.

It is of note, that similar telemedicine interventions were introduced in our facility regardless of the level of care, i.e., ambulatory, daycare, or inpatient interventions. This enabled ambulatory patients to receive more multidisciplinary treatment sessions during the COVID-19 outbreak than before, and daycare and inpatients to receive personally adjusted flexible programs in comparison to the more standardized interventions provided in regular times. The favorable effects of telemedicine were exemplified by the large number of patients who chose to continue with it even when face-to-face services were resumed.

Finally, we hope that the reported information of our case series can assist clinicians in their management of their ED patients and contribute to the limited literature on the treatment of adolescents with EDs during the still ongoing COVID-19 pandemic.

## Data Availability

Not applicable.

## Abbreviations

EDs: Eating disorders
AN: Anorexia nervosa
BN: Bulimia nervosa
BMI: body mass index
